# Comparing the immunosuppressive potency of naïve marrow stromal cells and Notch-transfected marrow stromal cells

**DOI:** 10.1186/1742-2094-8-133

**Published:** 2011-10-07

**Authors:** Mo A Dao, Ciara C Tate, Irina Aizman, Michael McGrogan, Casey C Case

**Affiliations:** 1 Research Department San-Bio Incorporated 231 South Whisman Road, Mountain View, 94041, USA; 2 Production Development Department San-Bio Incorporated 231 South Whisman Road, Mountain View, 94041, USA

## Abstract

**Background:**

SB623 cells are expanded from marrow stromal cells (MSCs) transfected with a Notch intracellular domain (NICD)-expressing plasmid. In stroke-induced animals, these cells reduce infarct size and promote functional recovery. SB623 cells resemble the parental MSCs with respect to morphology and cell surface markers despite having been in extended culture. MSCs are known to have immunosuppressive properties; whether long-term culture of MSCs impact their immunomodulatory activity has not been addressed.

**Methods:**

To assess the possible senescent properties of SB623 cells, we performed cell cycle related assays and beta-galactosidase staining. To assess the immunomodulatory activity of these expanded NICD-transfected MSCs, we performed co-cultures of SB623 cells or MSCs with either enriched human T cells or monocytes and assessed cytokine production by flow cytometry. In addition, we monitored the immunosuppressive activity of SB623 cells in both allogenic and xenogenic mixed lymphocyte reaction (MLR).

**Results:**

Compared to MSCs, we showed that a small number of senescent-like cells appear in each lot of SB623 cells. Nevertheless, we demonstrated that these cells suppress human T cell proliferation in both the allogeneic and xenogeneic mixed lymphocyte reaction (MLR) in a manner comparable to MSCs. IL-10 producing T cells were generated and monocyte-dendritic cell differentiation was dampened by co-culture with SB623 cells. Compared to the parental MSCs, SB623 cells appear to exert a greater inhibitory impact on the maturation of dendritic cells as demonstrated by a greater reduction in the surface expression of the co-stimulatory molecule, CD86.

**Conclusion:**

The results demonstrated that the immunosuppressive activity of the expanded NICD-transfected MSCs is comparable to the parental MSCs, in spite of the appearance of a small number of senescent-like cells.

## Introduction

There is an important need for stromal cell lines that support neural cells and the mesenchymal stem cell (MSC) line SB623, transfected with the Notch-intracellular domain (NICD), appear to meet these criteria. In cultures of embryonic cortical neurons, SB623 cells produce extracellular matrix proteins which enhance and maintain neurite outgrowth [1]. In neonatal hippocampal organotypic culture, SB623 cell-derived soluble trophic factors rescue neural cells subjected to oxygen-glucose deprivation [2]. In experimental Parkinson's disease, grafting of SB623 cells efficiently reverses the degeneration of dopaminergic neurons by promoting endogeneous neuronal cell recovery [3,4]. And in stable stroke animal models, transplantation of SB623 cells reduces infarct size and promotes behavioral improvement [5]. These studies validate one of the therapeutic applications of SB623 cells - to supply trophic factors for the endogenous neural cells after injury or disease.

Human marrow stromal cells are attractive for cell therapy because they can be obtained with minimal invasiveness and can be expanded in culture. However, as non-immortalized primary cells, MSCs have limited regenerative potential, committing to cellular senescence after extensive *ex vivo *manipulation [6,7]. A potential upside of senescent cells is their robust cytokine secretome profile which could be beneficial in tissue regeneration. A potential downside is that the senescent-associated-secretome profile is thought to be pro-inflammatory [8-10]. To date, intracerebral implantation of human SB623 cells in stroke-induced animals has not triggered any immunological adverse effect. Nevertheless, as SB623 cells are derived from MSCs that have undergone gene transfection and cell expansion in culture, we initiated the current study to determine whether SB623 cells display senescent-like properties. More importantly, we compare the immunomodulatory activity between SB623 cells and the corresponding parental MSCs. We demonstrate that SB623 cells, currently in a clinical trial for stable stroke (http://clinicaltrials.gov/ct2/show/NCT01287936), retain the immunosuppressive activity of standard MSCs despite the appearance of a small number of senescent-like cells.

## Materials and methods

### Production of MSCs and SB623 cells

MSC and SB623 cells were produced as previously reported [1,2]. Briefly, human adult bone marrow aspirates (Lonza, Walkersville, MD) were plated in growth medium - αMEM (Mediatech, Manassas, VA) supplemented with 10% fetal bovine serum (FBS) (Hyclone, Logan, UT), 2 mM L-glutamine and penicillin/streptomycin (both from Invitrogen, Carlsbad, CA) for three days to obtain the marrow stromal cell (MSC) monolayer. After two passages, a portion of the culture was cryopreserved as MSCs. The remaining cells (passage 2) were transfected with the pCMV-h*NICD1*-SV40- Neo^R ^plasmid using Fugene6 (Roche Diagnostics, Indianapolis, IN). After 7 days of selection with 100 μg/ml G418 (Invitrogen), the G418-resistant colonies were expanded and passed twice prior to cryopreservation as SB623 cells. This results in a uniformly transiently transfected population of MSCs.

### qPCR and qRT-PCR

Two days after transfection with pN2-NICD plasmid, cells were lysed and DNA or RNA purified using Qiagen's QIAAmp DNA or RNeasy mini kits (Qiagen, Valencia, CA), correspondingly, according to the manufacturer's protocols. Quantitative real time PCR or RT-PCR analyses were conducted using QuantiTect Probe PCR or RT-PCR kits, respectively, on Lightcycler (Roche).

For exogenous-NICD (eNICD) qPCR analysis, purified RNA-free DNA samples were used at 65 ng (10000 diploid human genomes) per reaction and eNICD gene copy numbers were determined using eNICD-DNA-specific Taqman assay (forward primer: TTGGTCTTACTGACATCCACTTTG, reverse primer CAGACACTTTGAAGCCCTCAG, exo-NICD-specific probe [6-FAM]CCCAGTTCAATTACAGCTCTTAAGGCTAGAG[BHQ1a-6FAM])). Amplification signals were compared to those of pN2-NICD plasmid serially diluted in human genomic DNA (Clontech, Mountain View, CA); results expressed in numbers of plasmids per one human diploid genome (plasmids/cell). For expression analysis of a NICD target gene, human Hes1 and GAPDH (control) Taqman assays (Applied Biosystems, Carlsbad, CA) were used. Normalized Hes1 expression levels are presented relative to levels in non-transfected cells.

### Phenotypic characterization by flow cytometry

For cell surface phenotyping, MSCs or SB623 cells were harvested with 0.25% Trypsin/EDTA (Invitrogen), washed in PBS/2% FBS, and re-suspended in 1 ml of PBS/2% FBS. Cells were then stained with fluorochrome-conjugated antibodies against CD29, CD31, CD34, CD44, CD45, CD73, CD90 (all from BD Biosciences, San Jose, CA) and CD105 (Invitrogen, Carlsbad, CA) for 15 minutes on ice. After one wash in PBS/2% FBS, cells were acquired using BD FACS Calibur. Analyses were done to assess the percentage of surface markers that are positive (CD29, CD44, CD73, CD90, and CD105) versus negative (CD31, CD34, and CD45) for mesenchymal cells using CellQuestPro program (BD Biosciences). To compare the density of specific surface molecule expression on MSCs versus SB623 cells, the delta mean fluorescent intensity (dMFI) was calculated - e.g., dMFI of CD44 = (MFI of CD44) - (MFI of IgG).

For intracellular protein detection of p16Ink4A and NICD, cells were fixed with 4% paraformaldehyde and permeabilized with PBS/0.1% TritonX-100. After two washes in PBS/2% FBS, cell pellets were resuspended in 200 ul of PBS/2% FBS and divided into two tubes, one for staining with phycoerythrin (PE)-conjugated IgG (control) and the other for staining with PE-conjugated p16Ink4A antibody (BD Bioscience) or PE-conjugated NICD antibody (eBioscience). For intracellular cytokine detection, cells were treated with BrefeldinA for six hours prior to harvest. After fixation and permeabilization, cells were incubated with fluorochrome-conjugated antibody against human GM-CSF (BD Bioscience), IL-1a (eBioscience, San Diego, CA), IL-6 (BD Bioscience), TGFβ1 (RnD Systems, Minneapolis, MN) for one hour followed by two washes in PBS/2% FBS. Acquisition and analysis of all samples were performed on BD FACS Calibur using CellQuestPro software.

### Cell proliferation measurement

To quantify viable cell expansion, one million MSCs or SB623 cells were plated on Day 0 and cell counts by trypan blue exclusion were done on Day 3. For cell cycle profile after culture, one million MSCs or SB623 cells were fixed in 70% ethanol overnight at 4°C. After two washes in PBS/2% FBS, cells were incubated in one ml of staining buffer (50 μg/ml propidium iodide, 50 μg/ml RNAse) (Sigma, St. Louis, MO) in PBS/2% FBS for 30 min in the dark. Acquisition and analysis were done using CellQuestPro program on the FL-2 linear channel. For cell cycle kinetics over 5 days in culture, MSCs and SB623 cells were labeled with 5 μM of 5-(and-6)-carboxyfluorescein diacetate (CFSE) (Invitrogen) for 2 min at room temperature prior to culture. Flow cytometry acquisition and analysis were done on the FL-1 log channel.

### Generation of monocyte-derived dendritic cells (Mono-DC)

Peripheral blood was obtained from healthy donors and mononuclear cells recovered from buffy coat preparations by Ficoll Paque (Amersham Pharmacia, Sweden) gradient separation. Mononuclear cells were re-suspended in RPMI/10%FBS and plated in a T-75 flask overnight. Non-adherent cells were discarded and the flasks were rinsed twice with PBS. Adherent monocytes were recovered using 0.25% trypsin/2 mM EDTA. Purity was assessed by staining with FITC-conjugated antibody against human CD14, a monocyte surface marker (Becton Dickinson) and was routinely shown to be > 90%.

For monocytic-to-dendritic cell differentiation assays, monocytes were cultured in RPMI-1640 (Mediatech) containing 10% FBS, 2 mM glutamine, 2 mM sodium pyruvate, 100 U/mL penicillin, 100 μg/mL streptomycin, 40 ng/mL granulocyte-macrophage colony stimulating factor (GM-CSF) and 20 ng/mL interleukin-4 (IL-4) (both from Peprotech, Rocky Hill, NJ) in the presence of MSCs or SB623 cells at a 10:1 monocyte to MSC or SB623 cell ratio. On Day 5, a subset of cultures were harvested by 0.25% trypsin/2 mM EDTA and stained with fluorochrome-conjugated antibodies against CD1a and CD14 (eBioscience). Data acquisition and analysis were done on the FACS Calibur using CellQuestPro software.

To assess the impact of MSCs and SB623 cells on the maturation of dendritic cells, monocyte-derived dendritic cells were generated in the presence of GM-CSF and IL-4. On Day 5, human TNF-α (10 ng/ml; Peprotech) was added to each well with or without MSCs or SB623 cells. As previous studies confirmed a role of cyclosporin A in hindering dendritic cell maturation [11], addition of cyclosporin A (1 μg/ml; Santa Cruz Biotechnology, Santa Cruz, CA) in the absence of either MSCs or SB623 cells was included as an internal control. On Day 7, cells were incubated with a fluorochrome-conjugated monoclonal antibody against human CD86 (BD Bioscience), a co-stimulatory molecule for priming and activating naïve and memory T cells and analyzed on the BD FACS Calibur using CellQuestPro.

### *Ex vivo *culture of human peripheral blood T cells

Human T cells were enriched from peripheral blood using the T-cell isolation kit (StemCell Technologies, Vancouver, Canada) according to the manufacturer's protocol. Enriched T cells were cultured in RPMI-1640/10% heat-inactivated FBS/pen/strep overnight prior to use. On Day -1, 10,000 MSCs or SB623 cells were plated per well of 96-well U-bottom plates. On Day 0 of the culture assay, 100,000 enriched T cells were transferred to each well with a pre-established MSC or SB623 cell monolayer. As an internal control, T cell cultures were maintained in the absence of MSCs or SB623 cells. On Day 7, a sub-optimal dose of 25 ng/ml of phorbol 12-myristate 13-acetate (PMA)/0.5 μM Ionomycin (both from Sigma-Aldrich) was added in the presence of BrefeldinA (eBioscience, 1:1000) for 6 hours prior to harvest for intracellular detection of interleukin-10 (IL-10) and interferon gamma (IFN-γ). For IL-17 producing TH17 cells, T cells were co-cultured with SB623 cells or MSCs in the presence of IL-23, or in the presence of IL-23 alone. After sub-optimal activation with PMA/Ionomycin in the presence of BrefeldinA, the cells were stained with fluorochrome-conjugated antibody against IL-17A (eBioscience) and analyzed by flow cytometry.

For regulatory T cell culture, human enriched T cells were co-cultured with MSCs or SB623 cells in the presence of human interleukin-2 (IL-2) (Peprotech, Rocky Hill, NJ) at a 10:1 T cells to MSC or SB623 cell ratio for 7 days followed by cell surface staining for CD4, a helper T cell marker and CD25, the IL-2 receptor alpha chain. For FoxP3 intracellular staining, cells were fixed and permeabilized with CytoFix/Perm (eBioscience). PE-conjugated antibody against FoxP3 (clone PCH101, eBioscience) was used at 1:50 dilution and flow cytometry analysis was done gating on lymphocytes. For assessment of constitutive IL-10 production, intracellular staining with fluorochrome conjugated antibody against IL-10 was performed without PMA/Io stimulation on Day 7.

### Mixed lymphocyte reaction (MLR)

Human allogeneic mixed lymphocyte reaction was established using peripheral blood from unrelated healthy volunteers. To obtain responder cells, T cell enrichment using a commercial T-cell rosette separation kit (Stem Cell Technologies) was done based on the manufacturer's protocol. Enriched T cells (= responders) were labeled with 5 μM of 5-(and-6)-carboxyfluorescein diacetate (CFSE) (Invitrogen) for 2 min at room temperature. CFSE-labeled lymphocytes were then plated in a 96-well U bottom plate at a concentration of 100,000 cells per 100 μl per well. To obtain stimulator cells, peripheral blood buffy coat mononuclear cells were recovered after Ficoll-density gradient centrifugation and red blood cell lysis buffer (Sigma-Aldrich) was added for 10 min at 37°C. 100,000 stimulator cells were added to a tube containing 10,000 MSCs or SB623 cells; and the mixed cells were then centrifuged and re-suspended at 110,000 mixed cells per 100 μl. 100 μl of stimulator/MSC cell mix or 100 μl of stimulator/SB623 cell mix was added to each well of CFSE-responder cells. To assess the activation state of T cells in the MLR, cells were harvested on Day 2 and stained with a fluorochrome conjugated antibody against CD69 (BD Bioscience), an antigen induced on activated T cells. To monitor cell proliferation kinetics of T cells in the MLR, cells were harvested on Day 7 and stained with PE-conjugated CD4 antibody (BD Bioscience). Flow cytometry data acquisition was done on BD FACS Calibur, gating on CD4+ lymphocyte gate, and analysis was done using CellQuestPro.

Xenogeneic MLRs were established using postnatal day 9 Sprague-Dawley rat glial mix cells as stimulators and human peripheral blood T cells as responders. Briefly, rat brains were harvested and triturated prior to treatment with 0.25% trypsin (Invitrogen) for 30 min. Cell suspensions were filtered through a 70 μM cell strainer and overlaid on Ficoll prior to density centrifugation. Glial mix cells were cultured in DMEM/F12 (Mediatech)/10%FBS/pen-strep for 14 days prior to use in the MLR. Xenogeneic MLRs were performed at a similar cell ratio as allogeneic MLRs (100,000 glial mix: 100,000 CFSE-labeled human T cells: 10,000 MSCs or SB623 cells) over a 5-day period. CFSE dilution of human CD3-gated T cells was assessed by flow cytometry.

### Statistics

Statistical assessments (SigmaStat, Systat Software, Chicago, IL) were made for SB623 or MSC groups to determine if there were differences either between those two groups or in some cases compared to the internal assay controls. To compare co-cultures with SB623 cells to those with MSCs (n = 3-6 matched lots), Tukey's pairwise comparisons were made. To compare co-cultures with SB623 cells or MSCs to internal experimental controls (when n>1), a general linear model ANOVA, followed by Tukey's pairwise comparisons was used. An alpha value of 0.05 was used to assess if the means were significantly different. Data are reported as mean ± standard deviation.

## Results

### Comparison of SB623 cells with the corresponding parental MSCs

SB623 cells were expanded from human MSCs after transfection with an *NICD*1-expressing plasmid - a process that takes eight to ten weeks in culture (Additional File 1A). Morphologically, SB623 cells retained the mesenchymal appearance similar to the parental MSCs. However, in each tested culture of SB623 cells, the frequency of beta-galactosidase-positive cells was higher than the parental MSC cultures, suggesting the presence of senescent cells in SB623 cell culture (Additional File 1B). qRT-PCR for the exogenous *NICD1 *gene and *Hes1*, a downstream target of Notch signaling, validated the high expression of exogenous *NICD1 *DNA and the induction of endogenous *Hes1 *transcript after transfection (Additional File 1C). Intracellular flow cytometry analysis of NICD1 confirmed its reduction over increasing passages consistent with transient transfection (Additional File 1D).

**Figure 1:**
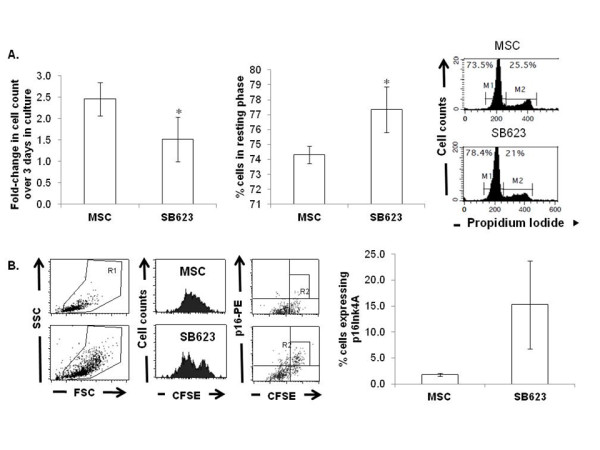
**SB623 cells proliferate more slowly and express more p16Ink4A than parental MSCs**. **A**) Cell proliferation assessed by cell counts (with trypan blue exclusion; left plot) and the percentage of G0/G1 cells measured by propidium iodide staining (middle plot) reveal a significant reduction in proliferation for SB623 cells compared to parental MSCs (*p < 0.05; n = 6); Far right shows representative FACS data for determining cell cycle profile. **B**) Cell division kinetics assessed by CFSE dilution and p16Ink4A protein by intracellular flow cytometry show a significant increase in p16Ink4A in SB623 cells versus parental MSCs (*p < 0.05); Representative FACS data on left and the mean expression for 4 different matched lots of MSC and SB623 cells on right.

To measure cell proliferation in culture, we plated one million MSCs or SB623 cells and used trypan blue exclusion to count the number of viable cells on Day 3. The results showed a higher number of viable cell counts for MSC culture than for SB623 cell culture, suggesting a lower proliferative index for SB623 cells. We next assessed the cell cycle profile by staining the cells with propidium iodide, a DNA intercalating dye, after fixation and permeabilization. We observed a significantly higher number of cells in G0/G1 resting phase (p < 0.05) in SB623 cultures, again suggesting reduced proliferation in SB623 cells (Figure 1A). Lastly, to monitor cell division kinetics over a 5-day growth culture, we opted for the use of CFSE, a cell permeable dye which is diluted with each cell division (Figure 1B). We noted a persistence of a small number of CFSE-high cells within each lot of SB623 cells and this was not observed in parental MSC where the vast majority of cells proliferated.

P16Ink4A is a negative cell cycle regulator and has been shown to be upregulated in human senescent MSC [6,7]. To determine whether the CFSE-high (low/no proliferating) cells express p16Ink4A, intracellular staining for the p16Ink4 protein was performed in CFSE-labeled cells after culture. The subpopulation of SB623 cells expressing p16Ink4A corresponded to the CFSE-high SB623 cells, consistent with the role of p16ink4A as a negative regulator of cell cycle entry (Figure 1B). Collectively, the results demonstrate that within each final lot of SB623 cells, there is a small number of non-proliferating cells.

**Figure 2:**
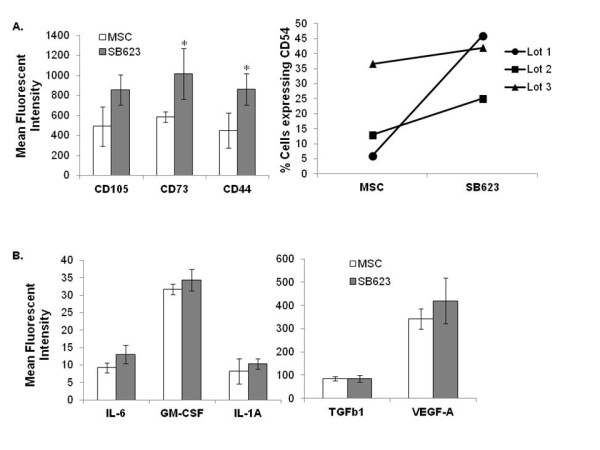
**Surface marker and cytokine expression profile of SB623 cells compared to parental MSCs**. **A**) Plots showing surface marker expression on SB623 cells and MSCs. Both SB623 cells and MSCs have >95% expression of CD44, CD73, and CD105, however, there is an increase in fluorescence intensity (measured by FACS) of these markers in SB623 cells compared to parental MSCs (*p < 0.05, n = 3, left plot); there is consistently higher expression of CD54 in SB623 cells versus matched lots of parental MSCs (right plot); **B**) Plots showing mean fluorescence intensity of IL-6, GM-CSF, IL-1A, TGFβ1, and VEGF-A (measured by intracellular antibody staining and flow cytometry) for 3 different matched lots of MSC and SB623 cells.

Phenotypically, SB623 cells expressed all the standard mesenchymal surface markers (CD73. CD105, CD29, CD44, CD106). However, CD44 and CD73 were expressed at significantly higher density per cell as shown by mean fluorescent intensity (Figure 2A). CD54, an inter-cellular adhesion molecule not commonly present on MSCs, was detectable on a small number of cells within each lot of SB623 cells.

In a previous study using cytokine array technology, Tate *et al*. characterized the secretome profile of SB623 cells compared to parental MSCs [2]. Here, using intracellular cytokine detection by flow cytometry, we compared the expression of trophic factors in SB623 cells and the corresponding parental MSCs by inhibiting protein secretion with BrefeldinA. The results demonstrate that although the amount of cytokines (IL-6, GM-CSF, IL-1a, VEGF-A, and TGFβ1) expressed varied between different lots, there was a general trend towards a small but detectable increase in IL-6 and GM-CSF intracellular protein expression in lots of SB623 cells compared to the corresponding lots of parental MSCs (Figure 2B).

### SB623 cells suppress T cell activation and proliferation comparable to parental MSCs in mixed lymphocyte reaction (MLR)

**Figure 3:**
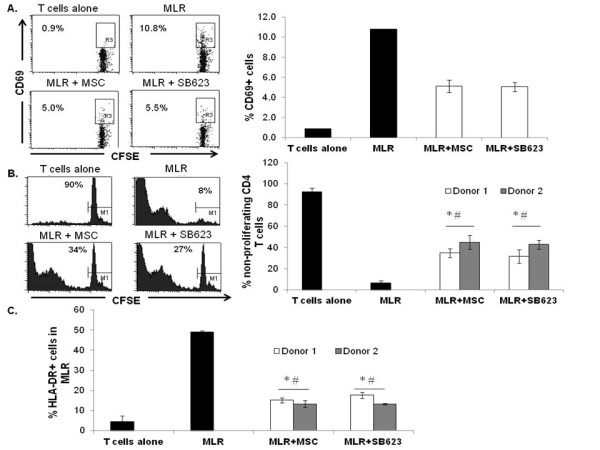
**MSC and SB623 attenuate T cell activation and proliferation in human allogeneic mixed lymphocyte reaction**. CFSE-labeled human enriched T cells plus allogeneic PBMCs were cultured with or without MSCs or SB623 cells. **A**) CD69 on day 2, **B**) non-dividing T cells (M1 gating on CFSE-high cells), and **C**) The percentage of cells expressing HLA-DR were assessed on day 5 by flow cytometry. Representative FACS data and the mean expression for 3 different matched lots of MSC and SB623 cells are shown. *p < 0.05 versus T cells alone; #p < 0.05 versus MLR.

Senescent cells have been shown to produce higher levels of pro-inflammatory factors than their younger counterparts [9]. Because we observed a small number of senescent-like cells within each lot of SB623 cells, we next compared the immunosuppressive activity of SB623 cells and the corresponding parental MSCs in the allogeneic mixed lymphocyte reaction. On Day 0, 10,000 SB623 cells or MSCs were added to each well of allogeneic mixed lymphocyte reactions (MLR), consisting of 100,000 CFSE-labeled peripheral blood enriched T cells and 100,000 peripheral blood mononuclear cells from unrelated donors. On Day 2, we assessed the induction of CD69, an early T cell activation marker. As shown in Figure 3A, the T cell activation marker, CD69, was robustly induced in the allogeneic MLR, validating the functionality of the assay. In the presence of SB623 cells, the percentage of CD4+ helper T cells expressing CD69 was reduced and this was comparable to the percent reduction seen with the parental MSCs. On Day 7, gating on CD4 immunostained T cells, we evaluated CFSE-dilution as an indicator of CD4+ helper T cell proliferation in MLR. As shown in Figure 3B, in the absence of MSC or SB623 cells, more than 80% of the CD4+ cells had proliferated. In the presence of SB623 cells, proliferation was significantly reduced (p < 0.05), comparable to the parental MSCs.

HLA-DR expression is known to be induced on activated T cells and on antigen presenting cells [12]. We assessed the percentage of HLA-DR-expressing cells within each MLR well as an additional measurement of cell activation. We demonstrated a significant reduction in HLA-DR-expressing cells when either SB623 cells or MSCs were included in the MLR (Figure 3C). These results from allogeneic MLR suggest that SB623 cells retain immunosuppressive activity comparable to their parental MSCs.

**Figure 4:**
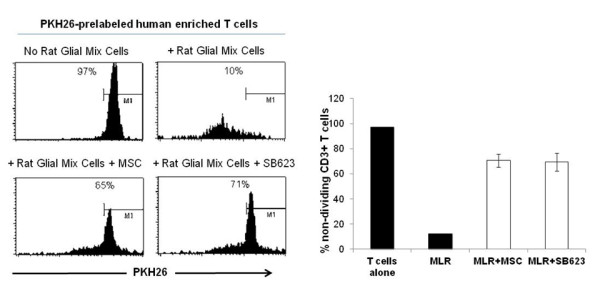
**MSCs and SB623 cells attenuate human T cell proliferation in xenogeneic mixed lymphocyte reaction**. PKH26 -labeled human CD3+ T cells plus rat mixed glial cells were co-cultured with or without MSCs or SB623 cells. PKH26 flow cytometry analysis gating on human CD3+ T cells was performed after 5 day co-culture with M1 gating on non-dividing T cells. Representative FACS data shown on top and the mean expression for 3 different matched lots of MSC and SB623 cells on bottom.

Transplantation of SB623 cells into rodents following experimental stroke has been performed via direct injection into recipient brain along with immunosuppressive drug administration [3-5]. To determine if SB623 cells and the parental MSCs can suppress T cell proliferation in a xenogeneic MLR, we isolated glial mix cells (astrocytes+microglia) to be used as stimulators for human CFSE-labeled T cells. By flow cytometry analysis gating on CD3, a marker present on all T cell subsets, we demonstrate that the addition of the parental MSCs as well as SB623 cells reduced the proliferation of human T cells in the xenogeneic MLR (Figure 4).

### SB623 cells support the generation of peripheral blood Treg-like cells comparable to parental MSCs

**Figure 5:**
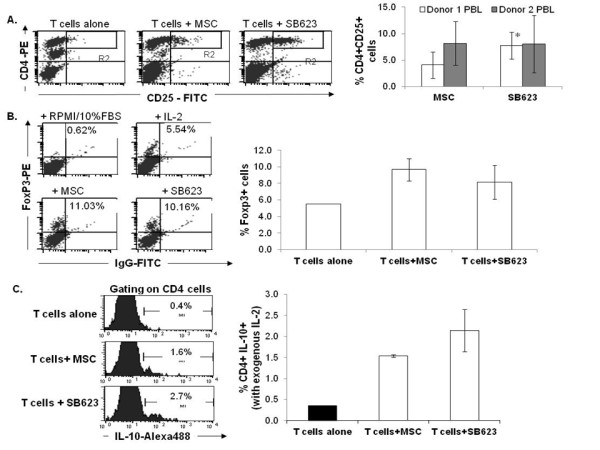
**Detection of CD25, FoxP3, and constitutive IL-10 in T cells co-cultured with MSCs or SB623 cells in the presence of exogenous IL-2**. Human T cells were co-cultured with MSCs or SB623 cells plus hIL-2 for 7 days. **A**) CD4 and CD25 surface expression with representative FACS data (left) and the mean expression for 5 different matched lots of MSC and SB623 cells (right); For "Donor 1 PBL", there is a significant increase in CD4+CD25+ cells when T cells were co-cultured with SB623 cells versus parental MSCs (*p < 0.05). **B**) The percentage of FoxP3-positive T cells as measured by intracellular staining with PE-conjugated antibody against FoxP3 followed by flow cytometry acquisition and analysis. The bar graphs represent the mean percentage of FoxP3-expressing T cells after co-culture without or with 3 different matched lots of MSC and SB623 cells. **C**) To assess the basal constitutive expression of IL-10, BrefeldinA (1:1000) was added during the last 6 hours of culture to inhibit the secretory pathway. By intracellular staining with a fluorochrome conjugated antibody against IL-10 and analyzed by flow cytometry. Shown here is a representative flow cytometry data data (left) and the mean expression for 3 different matched lots of MSC and SB623 cells (right) looking at the percentage of cells staining positive for intracellular IL-10 protein.

One of the mechanisms by which MSCs suppress immune activity is through the support for regulatory T (Treg) cell development [13-15]. IL-2, TGFβ1 and Notch ligands have all been shown to enhance regulatory T cell (Treg) differentiation [16-21]. To assess the potential of SB623 cells in supporting regulatory T cells in culture, we performed a 1:10 peripheral blood enriched T cells-to-MSCs or SB623 cell co-culture in the presence and absence of IL-2 over a 7 day period. CD25 expression on non-activated CD4+ T cells is commonly used as one of the identity markers for Tregs [22,23]. We therefore assessed the percentage of CD4+CD25+ cells within each culture and found significantly more CD4+CD25+ cells in co-cultures with SB623 cells than with MSCs for one of two blood donors tested (p < 0.05, Figure 5A). Another marker commonly used to identify Tregs is the transcription factor, FoxP3. By intracellular staining with a fluorochrome conjugated antibody against FoxP3 (clone PCH101) and analysis by flow cytometry, we demonstrate that the presence of MSCs and SB623 cells increased the detection of FoxP3-expressing T cells in the presence (>8% FoxP3+) of IL-2 (Figure 5B). And lastly, Tregs have been reported to constitutively produce IL-10. Therefore, by intracellular staining with fluorochrome conjugated antibody against IL-10, we assessed the percentage of T cells producing IL-10 in IL-2 treated T cell co-cultured with either MSCs or SB623 cells. The results demonstrate that MSCs and SB623 cells both enhanced the detection of IL-10 expressing T cells cultured in the presence of IL-2, compared to culture with only IL-2 (Figure 5C).

### SB623 cells alter the activated immune secretome profile similar to MSCs

**Figure 6:**
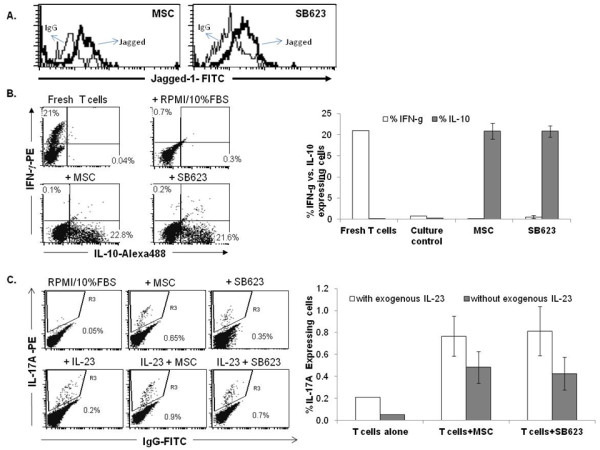
**MSCs and SB623 cells skew the "activated" T cell secretome profile**. Human T cells were co-cultured with MSCs or SB623 cells for 7 days in the absence of exogenous cytokines. To measure the activated T cell secretome profile, the cultures were stimulated with suboptimal doses of PMA/Ionomycin and Brefeldin A for an additional 6 hr prior to intracellular flow cytometry analysis of cytokines. **A**) Representative FACS analysis of Jagged-1 surface expression on MSC and SB623 cells prior to co-culture. **B**) Intracellular detection of IFN-γ and IL-10 of T cells after co-culture with or without MSCs or SB623 cells; Representative FACS data (left) and mean expression for 3 different matched lots of MSC and SB623 cells (right). **C**) Intracellular detection of IL-7A of T cells after co-culture with MSCs or SB623 cells in the presence or absence of IL-23. Negative controls include T cells cultured in RPMI/10%FCS alone and T cells cultured in the presence of IL-23 alone.

Independent studies suggest that MSCs skew the activated cytokine profile of immune cells, from a pro- to anti-inflammatory state [24,25]. Receptor ligands such as Notch ligand and TGFβ1 have immunomodulatory activity and can be presented by various environmental cells, including MSCs. As SB623 cells express the Notch ligand Jagged-1 (Figure 6A) and TGFβ1 (Figure 2), we performed additional cellular immune assays of human T cells co-cultured with either SB623 cells or MSCs followed by sub-optimal doses of PMA/Ionomycin in the presence of BrefeldinA on Day 7 to monitor the activated immune secretome profile by intracellular antibody staining for specific cytokines.

For Th17 cells, enriched T cells were co-cultured with either SB623 cells or MSCs in the presence or absence of exogenous IL-23 for 7 days. Intracellular detection of IL-17A expression by flow cytometry demonstrated a small percentage of IL-17A expressing T cells, averaging to less than 1.5% (Figure 6C). For Th1 cells, enriched T cells were co-cultured with either SB623 cells or MSCs in the absence of exogenous cytokines. As Th1 can secrete both IFN-γ and IL-10, we performed dual staining for these two cytokines to determine if the inclusion of SB623 cells skewed the activated immune secretome. We demonstrate that the inclusion of either MSCs or SB623 cells resulted in robust skewing of the activated immune secretome profile with more than 20% of the cells expressing IL-10 with less than 0.5% of the cells expressing IFN-γ in cultures that included either MSCs or SB623 cells.

### SB623 cells impede monocyte-to-dendritic cell differentiation in a manner comparable to parental MSCs

**Figure 7:**
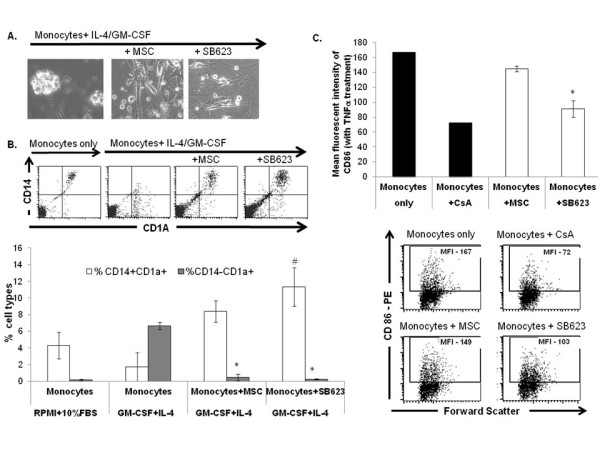
**MSCs and SB623 cells interfere with monocyte-DC differentiation and maturation**. Human monocytes were differentiated along the dendritic lineage with GM-CSF+IL-4, with or without MSCs or SB623 cells. **A**) Phase contrast images illustrating monocytic cell clustering; **B**) CD1A and CD14 staining on day 7. Representative FACS data (above) and mean expression for 3 different matched lots of MSC and SB623 cells (below); the percentage of cells co-expressing CD14 and CD1A after GM-CSF+IL-4 culture is significantly higher when monocytes were cultured with either MSCs or SB623 cells versus alone (#p < 0.05); the percentage of CD14-CD1A+ cells is significantly lower when differentiation culture included either MSCs or SB623 cells versus alone (*p < 0.05); **C**) CD86 surface expression after 2-day TNF-α treatment; Representative FACS data (left) and mean fluorescence intensity for 3 different matched lots of MSC and SB623 cells (right); there is a significant decrease in the mean fluorescent intensity of CD86 co-stimulatory molecule when monocytes were co-cultured with SB623 cells versus parental MSCs (*p < 0.05).

Another immunomodulatory property of MSCs lies in their ability to block monocyte differentiaton along the dendritic lineage [26-30]. By cytokine array, we previously identified IL-6 and VEGF as being secreted by SB623 cells [2]. Both of these cytokines have been shown to regulate dendritic cell differentiation and maturation [31-34]. To determine if SB623 cells can prevent monocytic differentiation to the dendritic lineage, we performed a 1:10 co-culture of SB623 cells with peripheral blood monocytes in the presence of IL-4 and GM-CSF. In parallel, we established co-cultures with the parental MSCs. After 7 days, phase contrast microscopy pictures were taken and each culture was stained with fluorochrome conjugated antibodies against CD14, a surface marker of monocytes, and CD1a, a surface marker of dendritic cells. As shown in Figure 7A, in the absence of SB623 cells or MSCs, dendritic cell clusters were readily visible. In contrast, such clusters were rarely seen in co-cultures with SB623 cells or MSCs. By flow cytometry, we noted that in the presence of IL-4 and GM-CSF, there was a conversion of CD14+CD1a+ dendritic cell precursors to predominantly CD14-CD1a+ dendritic cells. In contrast, when SB623 cells or parental MSCs were included in the monocyte-dendritic cell differentiation cultures, the transition was significantly reduced (Figure 7B). These results demonstrate that SB623 cells retain the ability to suppress monocytic-dendritic cell differentiation.

### SB623 cells impede dendritic cell maturation better than parental MSCs

Studies show that IL-6 can block dendritic cell maturation *in vivo *[33] while VEGF inhibits maturation in response to lypopolysaccharides (LPS) *in vitro *[34]. As shown in Figure 2, SB623 cells secrete both IL-6 and VEGF. To assess the ability of SB623 cells to dampen dendritic cell maturation, peripheral blood monocytes cultured for 7 days with GM-CSF and IL-4 were stimulated with TNF-α for an additional 48 hrs to promote maturation. SB623 cells or MSCs were added during this 48 hr stimulation. Two additional conditions - TNF-α alone or TNF-α + Cyclosporine A - were used as internal controls. At each endpoint, the expression levels of the T cell co-stimulatory molecule CD86 were assessed by flow cytometry (Figure 7C). Consistent with a previously published report [11], Cyclosporine A inhibited the induction of co-stimulatory molecule CD86, compared to conditions with TNF-α alone. Both SB623 cells and MSCs attenuated CD86 expression levels. Notably, SB623 cells were significantly more effective than MSCs (p < 0.05).

## Discussion

*Ex vivo *manipulation of MSCs has been shown to induce their cellular senescence [6,7] and senescent cells have been described to have a pro-inflammatory secretome [8-10]. Because SB623 cells are derived from *ex vivo *manipulated MSCs, we investigated the possible senescent onset in multiple lots of SB623 cells and compared the immunomodulatory activity of SB623 cells to that of the parental MSCs.

Morphologically, SB623 cells resemble their parental MSCs. Phenotypically, SB623 cells expressed all the standard MSC surface markers (CD90, CD105, CD29, CD44, CD73), although there was an increased density of CD44 and CD73. A small number of SB623 cells expressed surface CD54, an inter-cellular adhesion molecule serving as the ligand for LFA-1, the lymphocyte function-associated antigen. SB623 cells displayed a similar cytokine expression profile as parental MSCs. The effect of Notch in MSCs has been under much investigation. One study has implicated a function of Notch in promoting cellular senescence of rodent cells [35]. In our system, we transiently expressed *NICD *in human MSCs by DNA plasmid transfection. Analyzing for two senescent markers - beta-galactosidase positivity and p16Ink4A expression, we noted a small number of senescent-like cells within each lot of SB623 cells. From cell cycle profile and kinetics, we observed reduced proliferation in SB623 cultures compared to the parental MSCs. This reduced proliferation is most likely not mediated by exogenous *NICD *transient expression as we observed similar reduction in growth for MSCs transfected with an empty expression vector (data not shown). Therefore, we suspect that the small number of senescent-like cells within each lot of SB623 cells is a reflection of the extended time in culture (~2 months).

As noted above, some studies have highlighted the pro-inflammatory secretome of senescent cells [8-10]. To date, we did not observe immunological side effects from SB623 cell implantation in rats. Nevertheless, as we noted a small population of senescent-like cells within each lot of SB623 cells, we initiated various cellular immune assays to compare their immunomodulatory activity to parental MSCs in more detail. In an allogeneic mixed lymphocyte reaction (MLR), we demonstrated that similar to MSCs, SB623 cells attenuated the activation of CD4+ T cells as evident by reduction in CD69 (an early T cell activation marker) and HLA-DR (an activation marker on both T cells and monocytes). In experimental rodent stroke, intracerebral implantation of SB623 cells elicits functional recovery [5]. As the glial cells are among the common antigen presenting cells in the nervous system, we assessed the efficiency of SB623 cells in suppressing the proliferation of human T cells stimulated by rat glial mix cells. We demonstrate that SB623 cells elicited immunosuppressive activity in this xenogeneic MLR assay, comparable to parental MSCs.

In the context of immune modulation, MSCs have been reported to impact both the innate and acquired immune cells [25]. In a standard mono-dendritic cell differentiation assay with GM-CSF and IL-4, we demonstrate that the inclusion of SB623 cells in the mono-dendritic cell differentiation culture reduced the production of dendritic cells (CD1a+CD14-) to similar extent as the parental MSCs. In a 2-day TNF- α mediated dendritic cell maturation assay, the inclusion of SB623 cells reduced the density of CD86 co-stimulatory molecules, as measured by mean fluorescent intensity. Interestingly, the reduction in CD86 surface expression was significantly higher in the presence of SB623 cells than the parental MSCs. A recent study reported that activated MSCs secrete soluble TNF-α receptors which, in turn, attenuate systemic inflammation [36]. As TNF- α is commonly used to induce dendritic cell maturation, we hypothesize that a differential expression and/or secretion of TNF- α receptors between SB623 cells and MSCs could explain our current observations. Additional studies are warranted to address this possible underlying mechanism of action.

To assess the impact of SB623 cells on the acquired immune cells, we performed co-cultures of human enriched T cells with SB623 cells or MSCs and assessed the activated T cell secretome profile following stimulation with sub-optimal dosage of PMA/Ionomycin. By intracellular staining with antibodies against IL-10 and IFN-γ, we observed a robust skewing in the activated immune secretome profile with more than 95% of cells expressing IL-10 and less than 5% expressing IFN-γ. The detection of predominantly IL-10 expressing cells is in line with previous reports that MSCs promote the anti-inflammatory secretome of T cells. The lack of IFN-γ production was unexpected since Th1 cells are known to produce both IFN-γ and IL-10, not just IL-10 alone. However, IL-10 has been shown to downregulate IFN-γ production [37-39]. It is therefore possible that in the presence of SB623 cells or MSCs, the level of IL-10 produced was high enough to form a negative feedback loop on the production of IFN-γ. A few studies have highlighted the production of IL-10 from Th1 T cells as a "self-control" mechanism [40,41] and Notch activation has been associated with this process [42]. TGFβ1 and IL-6, both of which are known to be produced by MSCs, have a role in Th17- and Th1- T cell development. To determine if SB623 cells impact the number of Th17-T cells, we performed co-cultures of T cells with SB623 cells or MSCs in the presence or absence of IL-23, a cytokine supportive of human Th17-T cell development. By intracellular staining with an antibody against IL-17A, we detected an average of less than 1.5% Th17-T cells, with no significant differences between co-cultures with SB623 cells or the parental MSCs. While it is surprising to see a positive impact of marrow stromal cells on Th17 cell number in culture, one study to date has highlighted this property of fetal marrow stromal cells [43]. It is also important to note that the percentage of IL-17-producing T cells is relatively low compared to the percentage of IL-10 producing T cells and thus, may explain why transplantation of MSC elicits an overall immunosuppressive outcome [24,25].

Another immunomdulatory property of TGFβ1 and Notch ligands is in the regulation of regulatory T cells (Tregs) [13,16,21]. As both TGFβ and Notch ligand are expressed by SB623 cells and the parental MSCs, we next investigated the impact of SB623 cells on T cells cultured in the presence of IL-2, a cytokine important in Treg cell development. In the presence of SB623 cells, we observed a higher percentage of cells expressing surface CD25, the IL-2 receptor alpha chain. Although CD25 is commonly used to identify Tregs [22,23], this surface marker is also present on activated T cells [44]. An alternate common marker for Tregs is the forkhead family transcription factor FoxP3[45]. By intracellular staining with an antibody against FoxP3, we demonstrated a higher percentage of FoxP3+ cells when SB623 cells or MSCs were included. As FoxP3 is not exclusively expressed in Tregs [46], we measured the intracellular expression of IL-10, a cytokine constitutively produced at low levels by Tregs [47,48]. We consistently detected a small but higher percentage of CD4+ T cells expressing IL-10 in cultures with SB623 cells or MSCs compared to the controls. These results suggest that SB623 cells, like the parental MSCs, may have a role in supporting T cells having various features of regulatory T cells.

SB623 cells are derived from NICD-transfected MSCs and expanded in the absence of exogenous cytokines. As such, SB623 cells retained the standard phenotypes and morphology of conventional MSCs. Compared to the early passage MSCs, SB623 cells contained a small number of senescent-like cells as a result of cell expansion *in vitro*. Nevertheless, we show here that SB623 cells effectively suppressed T cell proliferation in MLR and modulated the T cell secretome profile as efficiently as the parental MSCs. The current study demonstrate that the transient overexpression of exogenous *NICD *with subsequent expansion of human MSCs preserved, and in some cases increased, their immunosuppressive activity.

## Disclosure

All authors are employees of San-Bio Inc.

## Authors' contributions

MD conceived and designed the study, performed immunoassays and flow cytometry, analyzed and interpreted data, and wrote the manuscript. CCT participated in data analysis and interpretation, performed statistical analysis, and edited the manuscript. IA prepared DNA and RNA samples, performed PCR, analyzed and interpreted PCR data. MM designed and coordinated the production of MSCs and SB623 cells. CCC directed the study, designed PCR primers for the detection of exogenous NICD, and helped with data interpretation. All authors read and approved the final manuscript.

## Supplementary Material

Additional file 1 **Production and characterization of SB623 cells**. A) Representative illustration of SB623 cell production. Marrow stromal cells were established at passage 2, followed by plasmid transfection and drug selection, and ending with cell expansion for two additional passages to generate the end product, SB623 cells. B) Beta-galactosidase staining of MSCs and SB623 cells culture. MSCs of passage 2 and SB623 cells plated for 48 hr in growth medium were stained with a commercial beta-galactosidase staining kit (Cell Signaling) according to manufacturer's protocol. C) Assessment of exogenous NICD DNA and endogenous Hes1 (downstream target of Notch) transcript in NICD-transfected MSC. D) Flow cytometric analysis for the percentage of NICD expressing cells during SB623 cell production. At different passages after transfection and selection, cells were harvested using 0.25% Trypsin, fixed with paraformaldehyde, and permeabilized with 0.1% Triton-X100. Samples were stained either with a fluorochrome-conjugated IgG or a fluorochrome-conjugated antibody against NICD protein. Cell acquisition and analyses were done on the BD FACS Calibur using CellQuestPro software. Click here for file
