# Supplementary figures and images for: Comparing the immunosuppressive potency of naïve marrow stromal cells and Notch-transfected marrow stromal cells
Source: J Neuroinflammation. 2011 Oct 7;8:133. doi: 10.1186/1742-2094-8-133 (PMC3228829; doi:10.1186/1742-2094-8-133)

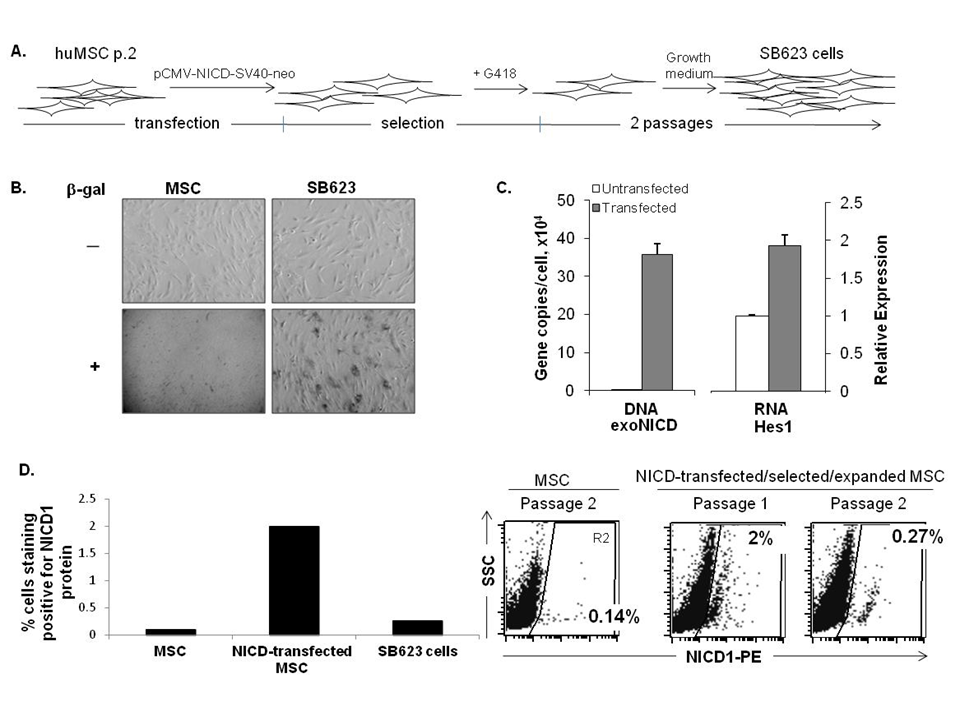

Supplement: Additional file 1 — Production and characterization of SB623 cells. A) Representative illustration of SB623 cell production. Marrow stromal cells were established at passage 2, followed by plasmid transfection and drug selection, and ending with cell expansion for two additional passages to generate the end product, SB623 cells. B) Beta-galactosidase staining of MSCs and SB623 cells culture. MSCs of passage 2 and SB623 cells plated for 48 hr in growth medium were stained with a commercial beta-galactosidase staining kit (Cell Signaling) according to manufacturer's protocol. C) Assessment of exogenous NICD DNA and endogenous Hes1 (downstream target of Notch) transcript in NICD-transfected MSC. D) Flow cytometric analysis for the percentage of NICD expressing cells during SB623 cell production. At different passages after transfection and selection, cells were harvested using 0.25% Trypsin, fixed with paraformaldehyde, and permeabilized with 0.1% Triton-X100. Samples were stained either with a fluorochrome-conjugated IgG or a fluorochrome-conjugated antibody against NICD protein. Cell acquisition and analyses were done on the BD FACS Calibur using CellQuestPro software. [file 1742-2094-8-133-S1.TIFF]
